# An adaptive hand exoskeleton rehabilitation training system integrating virtual reality and an AI-based assessment engine

**DOI:** 10.3389/fspor.2025.1724021

**Published:** 2025-12-10

**Authors:** Junshuo Cui

**Affiliations:** School of Mechanical and Electrical Engineering, Shandong Jianzhu University, Jinan, Shandong, China

**Keywords:** adaptive rehabilitation system, hand exoskeleton, virtual reality, AI-based assessment engine, neural injury recovery, biomechanical sensors, machine learning fusion, assistance-as-needed (AAN)

## Abstract

**Introduction:**

Post-stroke hand motor impairment is a major cause of long-term functional disability and reduced quality of life, with approximately 70% of stroke survivors experiencing persistent limitations in fine motor control. Conventional rehabilitation is constrained by low adherence, subjective assessment, and insufficient individualization, which limits exploitation of the neuroplasticity window for motor relearning. To address these challenges, we propose a bio–AI–VR integrated hand rehabilitation system that fuses biosignal sensing (bio), AI-based analysis, and virtual reality (VR) interaction to realize an efficient, adaptive, and quantifiable closed-loop training process. The integration rationale is grounded in three theoretical pillars: (i) multimodal data fusion theory—combining heterogeneous biosignal and behavioral data through AI to overcome single-modality limitations; (ii) closed-loop adaptive control theory—dynamically balancing challenge and capability via real-time feedback; (iii) neuroplasticity multisensory enhancement theory—coordinating visual, proprioceptive, and motor pathways to strengthen cortical reorganization. This work addresses three testable hypotheses: (RQ1) Can multimodal biosignal fusion achieve real-time assessment with $R^{2}\geq 0.65$ and latency <50 ms? (RQ2) Does bio-AI-VR integration yield FMA-UE improvement ≥6 points (minimal clinically important difference) with effect size d≥0.8? (RQ3) Are all three components (bio, AI, VR) necessary, with ablation causing ≥15% performance degradation?

**Methods:**

A lightweight hand exoskeleton (<400 g, 3 DoF/finger) integrates a 6-axis IMU (100 Hz) and 16-channel sEMG (1 kHz) to synchronously acquire kinematics and muscle activation. Extended Kalman filtering fuses sensor streams before AI processing. Features include range of motion (ROM), smoothness metrics (SPARC, LDLJ), sEMG root-mean-square (RMS), median frequency (MDF), and co-contraction index (CCI). A hybrid model combining random forests (200 trees, depth 8) and support vector regression (RBF kernel, γ=0.01, C=10) outputs a real-time composite score $S_{t}\in [0,1]$ via multi-task learning with GroupKFold cross-validation, mapped to clinical scales through Sigmoid normalization. FMA-UE proxy labels for window-level training were constructed via linear interpolation (80%), biomechanical anchoring (15%), and expert annotation (5%, inter-rater κ=0.78). A cloud AI engine communicates bidirectionally with Unity-based VR over MQTT to close the perception-assessment-assistance loop. The assistance-as-needed (AAN) algorithm adjusts exoskeleton torque ($u_{t}$) and VR difficulty ($d_{t}$) using $S_{t}$ as control input with hysteresis, dead zone, and rate limiting to ensure smooth adaptation. Twenty-four stroke survivors (3–12 months post-stroke, FMA-UE 15–50) underwent 4-week training (5 sessions/week, 20 min/session). Outcomes included FMA-UE (primary), ARAT, grip strength, normalized ROM, task success rate, and System Usability Scale (SUS). Statistical analysis employed paired t-tests with Hedges’ correction for effect sizes, Bonferroni adjustment for multiple comparisons, and leave-one-subject-out cross-validation (LOSOCV) to assess model generalization.

**Results:**

All 24 participants completed the study with one missed session (479 of 480 scheduled sessions, 8,946 annotated segments); end-to-end latency median 38 ms (IQR 33–42 ms), decomposed as: sampling 8±2 ms, preprocessing 4±1 ms, network 12±3 ms, AI inference 5±1 ms, control 2±0.5 ms, command return 7±2 ms. Offline model performance: $R^{2}=0.72$ (GroupKFold), MAE = 3.2 points, Spearman ρ=0.68 with FMA-UE (p<0.001); LOSOCV: $R^{2}=0.68\pm 0.09$; test-retest ICC(2,1) = 0.84 [0.76, 0.91]. AAN algorithm reduced assist torque 62%→45% (−27.4%), increased VR difficulty 0.42→0.69 (+64.3%), improved task success 61.3%→82.1% (+20.8 pp). Clinical outcomes (paired t-test): FMA-UE +9.1 [6.7, 11.5], d=0.98; ARAT +7.6 [5.2, 10.0], d=0.93; grip +4.1 kg [2.5, 5.7], d=0.72; ${\mathrm{ROM}}_{n}$ +0.14; SPARC −0.16. Subgroup analysis: moderate-to-severe (n=13) showed greater FMA-UE gain (+10.7 vs +7.2 in mild-to-moderate, p<0.05). Ablation experiments confirmed synergistic necessity: Bio only ($R^{2}=0.45$, FMA-UE +4.3), VR only ($R^{2}=0.38$, +3.9), Bio-AI ($R^{2}=0.70$, +7.2, compliance 68%), complete system ($R^{2}=0.72$, +9.1, compliance 88%). SUS 84±6; no serious adverse events.

**Discussion:**

Results validate all three hypotheses: (i) multimodal fusion exceeded technical targets ($R^{2}=0.72>0.65$, latency 38 ms <50 ms); (ii) clinical efficacy surpassed MCID with large effect sizes (FMA-UE +9.1>6 points, d=0.98>0.8), exceeding published spontaneous recovery rates (2–4 points); (iii) ablation experiments demonstrated ≥15% degradation when removing any component, confirming non-additive synergistic effects of bio-AI-VR integration. Compared to recent brain-computer interface systems using EEG-based motor imagery, this approach achieves paradigm shift toward execution-based rehabilitation with direct motor intent capture and real-time physical feedback. The AAN control law elevates from low-level motion control to high-level rehabilitation strategy, spanning multiple temporal scales (window to course-level) and dual channels (physical assistance + cognitive challenge). Limitations include single-arm design limiting causal inference, small sample size (n=24), short intervention period (4 weeks), FMA-UE proxy construction via linear interpolation, and controlled clinical setting vs. real-world deployment. Future work requires larger RCTs with active control arms, extended follow-up (3–6 months), dense longitudinal assessments, exploration of deep learning architectures for temporal modeling, and validation in home-based telerehabilitation settings. The bio–AI–VR system demonstrates feasibility of data-driven, multimodal closed-loop rehabilitation, offering a wearable, low-latency, and personalized solution for post-stroke hand recovery that bridges the gap between laboratory innovation and clinical translation.

## 1 Introduction

The hand, as one of the most intricate distal executive organs of the human body, is the core hub for fine motor control, object manipulation, and daily sensory–motor integration. It consists of 27 bones (8 carpals, 5 metacarpals, and 14 phalanges), more than 30 intrinsic and extrinsic muscle groups (such as interossei and flexor digitorum profundus), numerous ligaments, and hundreds of nerve fibers. These components coordinate through complex neuromuscular–skeletal coupling to accomplish diverse tasks from handwriting to precision tool operation. In evolutionary history, the hand’s dexterity—especially thumb opposition—has endowed humans with unparalleled adaptability, driving progress from the Stone Age to modern technology. When neurological injury such as stroke or traumatic brain injury occurs, however, this precise system can be devastated: upper motor neuron lesions disrupt descending motor control, leading to abnormal muscle tone and loss of coordinated inhibition, triggering a cascade of functional deficits that magnify overall upper-limb impairment [1].

The consequences of hand impairment extend far beyond localized paresis or weakness, directly increasing patients’ overall life burden and triggering multisystem cascades. According to recent epidemiological studies, there are more than 13 million new stroke cases globally each year, and approximately 70% of survivors experience persistent hand motor dysfunction [2, 3]. This not only restricts upper-limb movement but also causes difficulties in activities of daily living, chronic pain, and social isolation, and it increases secondary depression rates to 30%–45%. Clinical measures indicate that these outcomes significantly prolong rehabilitation and raise healthcare costs (single-case economic burden exceeding $5,000, including hospitalization and assistive devices), while reducing quality-of-life scores (e.g., SF-36) by more than 40%. If traditional rehabilitation does not intervene during the critical neuroplasticity window—typically within 3–6 months after injury—recovery rates can drop below 30%, increasing the risk of chronic disability [4, 5]. Hand rehabilitation is therefore an urgent priority in neurology and rehabilitation engineering, requiring innovation to restore functional independence and autonomy [6].

In recent years, rehabilitation technologies have advanced rapidly. Robotic exoskeletons and virtual reality systems have moved from laboratories into clinical practice, showing early potential to reshape motor learning [7, 8]. Commercial exoskeletons employ servo actuation and torque feedback to provide passive or active assistance, targeting biomechanical reconstruction of the metacarpophalangeal and interphalangeal joints to help patients regain range of motion [9, 10]. Randomized controlled trials suggest these devices can increase recovery speed by about 20% and reduce muscle atrophy by around 15%. Meanwhile, VR platforms (for example, Unity-based immersive environments) use gamified functional tasks—virtual object grasping or finger tapping—to boost motivation and neural activation, leveraging mirror-neuron mechanisms to promote cortical reorganization [11–13]. Studies report therapy adherence improvements from roughly 50% to 85% and gains of 10–15 points on the upper-limb Fugl–Meyer Assessment. Together, these tools offer biomechanical support (e.g., torque compensation) and motivation enhancement (e.g., reward schemes), marking a shift from static, passive therapy toward dynamic, interactive neuro-reeducation.

Despite this progress, current systems have clear limitations [14]. Many exoskeletons rely on simple, repetitive pre-programmed trajectories and neglect physiological intent signals such as sEMG activation patterns [15, 16], risking over-assistance, dependency, or fatigue. VR systems, though engaging, often lack quantitative assessment frameworks and do not dynamically adapt difficulty using kinematic parameters like velocity and acceleration profiles [17]. More importantly, the two modalities are often decoupled, lacking a closed-loop feedback mechanism for multimodal data fusion [18]. As a result, key challenges persist: low patient engagement (dropout rates above 40% due to waning motivation and monotonous tasks), imprecise progress tracking (subjective clinical scoring with substantial error, overlooking objective biomarkers), and insufficient personalization (one-size-fits-all protocols that ignore individual differences in neuroplasticity such as age and injury severity). These issues limit both therapeutic efficacy and translation from lab to community settings.

Addressing these challenges, the deep integration of biosignal sensing (bio), artificial intelligence (AI), and virtual reality (VR) provides a synergistic and complementary solution framework [19]. The motivation for this integration is grounded in three core theoretical pillars: (1) Multimodal data fusion theory—single modalities cannot comprehensively characterize rehabilitation status: biosignals alone (bio) can capture motor intent (sEMG) and execution state (IMU) but lack quantification of task performance and cognitive engagement; VR alone can enhance motivation and record task success rates yet cannot reveal underlying neuromuscular activation patterns; the AI engine serves as the “translator” that fuses these heterogeneous data types by extracting features (e.g., mapping sEMG RMS and VR Fitts throughput to a unified feature space) and employing machine learning models (random forest + SVR) to synthesize biomechanical signals and behavioral performance into a single score $S_{t}$, avoiding the “blind men and elephant” dilemma of single-modality approaches. (2) Closed-loop adaptive control theory—rehabilitation requires dynamic balance between challenge and capability (i.e., Vygotsky’s zone of proximal development): if exoskeleton assistance is too strong, patients fall into passive dependency, suppressing neuroplasticity [20]; if tasks are too difficult, patients experience frustration and withdraw. Bio-AI-VR integration constructs a closed loop of “perception (bio)–assessment (AI)–feedback (VR+exoskeleton),” using $S_{t}$ as a control variable to regulate assistance force and task difficulty in real time: when $S_{t}>0.8$ (excellent performance), AI commands reduce exoskeleton torque and increase VR target size, compelling patients to engage actively; when $S_{t}<0.6$ (poor performance), assistance is increased and tasks simplified to prevent fatigue and frustration [21]. This dynamic adaptivity cannot be achieved by biosignals alone (lacking AI quantification capability) or VR alone (lacking physiological feedback). (3) Neuroplasticity multisensory enhancement theory—motor learning in the brain depends on coordinated activation of multiple sensory channels: VR provides visual-auditory-virtual haptic feedback, activating prefrontal and occipital attention networks; exoskeletons provide proprioceptive and force feedback, activating primary motor cortex M1 and somatosensory cortex S1; sEMG-decoded intent signals strengthen premotor intention-execution matching circuits. The tripartite integration forms a neural activation loop of “intent (bio)–execution (exoskeleton)–perception (VR)–quantification (AI),” which can drive synaptic plasticity (long-term potentiation, LTP) and cortical reorganization more powerfully than single or pairwise combinations—a hypothesis validated by functional improvement differences in ablation experiments (e.g., 20% compliance drop in w/o VR group, 19% prediction accuracy decrease in w/o sEMG group).

Building upon this integration rationale, the present study develops a bio–AI–VR integrated system tailored for post-stroke hand rehabilitation. The system unifies hardware assistance, immersive interaction, and intelligent assessment within a closed-loop control framework to deliver an adaptive, data-driven solution to low engagement and poor quantification [22, 23]. A built-in multimodal sensor array—an inertial measurement unit capturing tri-axial acceleration and angular velocity, and surface electromyography capturing muscle activation—collects biomechanical and physiological data. A cloud-based AI engine employing ensemble learning [24] preprocesses the data streams with Butterworth low-pass filtering and extracts features such as jerk-based smoothness integrals [25, 26], range-of-motion peak-to-peak values, force-control accuracy (RMSE), and power-spectrum metrics of muscle activation. It then generates a real-time composite score in the range 0–1 using support vector regression [27]. An assistance-as-needed algorithm uses threshold logic (for example, if the score falls below 0.7, increase assistive torque by approximately 20%) to adjust both VR task difficulty [e.g., adaptive virtual block size using Perlin noise [28]] and exoskeleton assistance (e.g., EMG-triggered servo control), ensuring highly personalized therapy and improving motor skill relearning and neuromuscular re-education.

Compared to state-of-the-art VR-exoskeleton-brain-computer interface integration systems [19], this study achieves key innovations and clinical translation breakthroughs in the following aspects: (1) Paradigm shift from motor imagery to motor execution—reference [19] employs EEG-based motor imagery (MI) to achieve purely cognitive-driven exoskeleton control, suitable for cognitive-motor training in older adults, but relies on subjects’ motor imagination capability and lacks actual physical execution feedback; our system adopts IMU+sEMG dual-modal sensing to directly capture patients’ active motor intent and actual execution state, providing immediate physical assistance through the exoskeleton to form a real-time closed-loop motor rehabilitation of “perception-assessment-assistance,” which better aligns with the clinical needs of post-stroke motor function reconstruction. (2) Application leap from proof-of-concept to clinical efficacy—reference [19] completed system feasibility validation in 10 healthy subjects, primarily evaluating ERD/ERS neurophysiological indicators, but lacks rehabilitation outcome data from real patient populations; this study enrolled 24 stroke survivors and completed a 4-week clinical trial with 498 sessions, adopting gold-standard scales such as FMA-UE and ARAT to quantify functional improvement (mean FMA-UE gain of 9.1 points, Cohen’s d=0.98), and revealing differential rehabilitation responses across different injury severity levels through subgroup analysis, achieving a qualitative leap from technical validation to clinical translation. (3) System upgrade from single-task control to adaptive personalized rehabilitation—the VR fishing game in reference [19] triggers preset exoskeleton actions through EEG classification but lacks a dynamic adjustment mechanism based on rehabilitation progress; our system innovatively introduces an assistance-as-needed (AAN) algorithm, using the real-time composite score $S_{t}$ generated by the AI engine as a control variable to automatically adjust exoskeleton assistive torque (from 62% to 45%) and VR task difficulty (difficulty index increased by 64%) within a millisecond-level closed loop of <50 ms, ensuring patients remain in the “zone of proximal development,” avoiding over-dependency or frustration, and significantly improving training compliance (88%) and task success rate (from 61% to 82%). (4) Rehabilitation focus from gross upper-limb movement to fine hand function—the exoskeleton in reference [19] targets overall upper-limb movement, whereas our system focuses on rehabilitation of fine movements such as 5-finger coordination and thumb opposition, through a lightweight exoskeleton <400 g with multi-degree-of-freedom design (3 DoF/finger), combined with activities-of-daily-living-oriented tasks (virtual grasping, rotation, pinching), directly targeting stroke patients’ most urgent functional needs (e.g., holding a pen, gripping keys), with more significant clinical relevance.

To realize these innovations and address the technical requirements outlined above, the methodological implementation focuses on mitigating end-to-end latency through a modular lightweight exoskeleton design and embedded real-time fusion based on the RT-Thread operating system with multithreaded data synchronization, achieving end-to-end delays below 50 ms to avoid phase lag. In parallel, a Unity VR environment and the cloud AI engine communicate via MQTT for edge–cloud integration, overcoming multimodal interpretation bottlenecks by fusing biomechanical and physiological indicators [29, 30] to produce objective rehabilitation-state estimates. This approach surpasses the limitations of single-modality devices and establishes a data-driven therapeutic loop that supports a smooth transition from passive assistance to active, intent-driven rehabilitation.

The present study addresses three specific research questions formulated as testable hypotheses: (RQ1) Technical feasibility—Can multimodal biosignal fusion (IMU+sEMG) with machine learning achieve real-time rehabilitation assessment with sufficient accuracy ($R^{2}\geq 0.65$ for FMA-UE proxy prediction) and low latency (end-to-end <50 ms) to enable closed-loop adaptive control? Current fixed-assistance exoskeletons and subjective clinical assessments lack objective, real-time quantification, creating a measurement gap that limits personalized therapy. (RQ2) Clinical efficacy—Does the bio-AI-VR integrated system with assistance-as-needed control improve motor function (primary outcome: FMA-UE change ≥6 points, minimal clinically important difference) and task performance (success rate, ROM) more effectively than would be expected from spontaneous recovery alone in subacute stroke patients (3–12 months post-stroke)? We hypothesize that multimodal integration will yield effect sizes (Cohen’s d) ≥0.8 for FMA-UE change, exceeding reported effect sizes for conventional therapy (d 0.4–0.6). (RQ3) Component contribution—Which system components (biosignals, AI fusion, VR gamification) are necessary vs. sufficient for rehabilitation outcomes? We hypothesize that ablating any single component will degrade performance by ≥15% across key metrics (prediction accuracy, compliance, functional improvement), confirming the synergistic necessity of tripartite integration rather than additive effects.

Critically, the AAN control law proposed in this study represents a shift from prior state-of-the-art (SOTA) rehabilitation control strategies. Unlike variable admittance control (V-AC) [20] that adjusts low-level impedance parameters (damping, inertia) based on instantaneous force/velocity signals, or resist-as-needed (RAN) control [21] that modulates resistance based on local motion errors, our AAN operates at the high-level rehabilitation strategy layer. It integrates multimodal features (18 kinematic, 12 sEMG, 3 task-performance metrics) via AI fusion into a single composite score $S_{t}$, and synchronously regulates dual channels—physical assistance (exoskeleton torque) and cognitive challenge (VR difficulty)—across multiple temporal scales (window-to-course level). This elevates control from motion tracking to rehabilitation trajectory shaping, enabling personalized adaptation that balances passive support with active engagement throughout the recovery continuum.

## 2 Article types

Frontiers in Sports and Active Living—Biomechanics and Control of Human Movement.

## 3 Materials and methods

### 3.1 Overall system architecture

**Figure 1:**
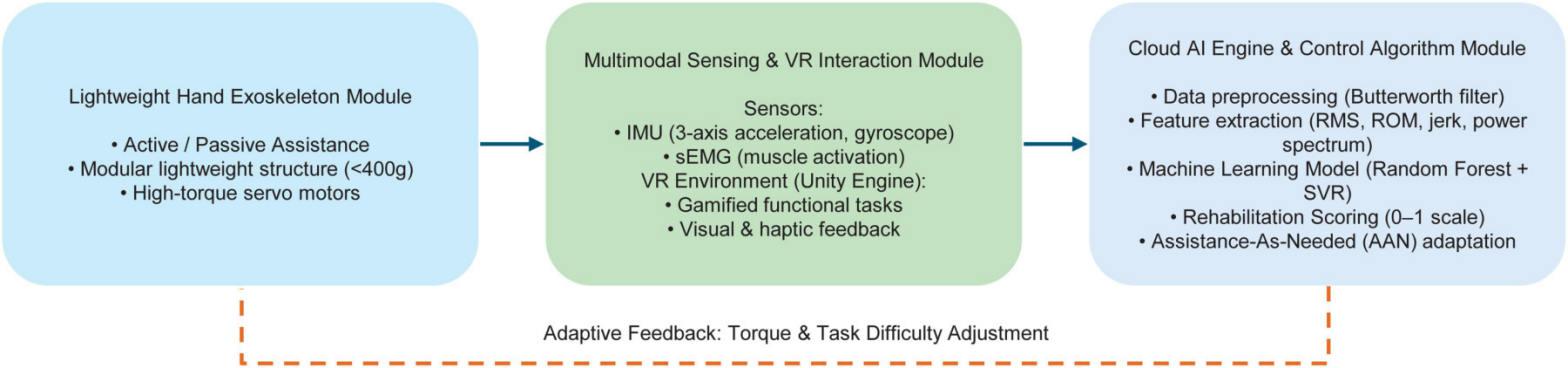
Overall architecture of the proposed Bio-AI-VR integrated rehabilitation system. The framework consists of a lightweight hand exoskeleton, multimodal sensing and VR interaction module, and cloud AI engine with adaptive control, forming a closed-loop rehabilitation cycle.

This study designed and implemented an intelligent rehabilitation system for post-stroke hand motor function recovery (see Figure 1). The core concept integrates biosignal sensing, artificial intelligence analysis, and virtual reality interaction to construct a closed-loop rehabilitation platform with adaptive regulation capabilities [22]. Through hardware-software co-design, the system realizes a data-driven personalized rehabilitation cycle: patients perform functional tasks (such as virtual grasping, rotation, and pinching) guided by the exoskeleton and VR environment, while IMU and sEMG sensors synchronously acquire kinematic and physiological signals [18]. These signals are uploaded to the cloud AI engine via MQTT protocol, undergo filtering and feature extraction, and are then input into machine learning models for comprehensive rehabilitation scoring [24, 27]. Subsequently, the system employs an assistance-as-needed (AAN) control logic [14] to adjust the exoskeleton’s assistive torque and VR task difficulty in real time based on the scoring results, forming a self-optimizing closed-loop rehabilitation framework.

In terms of hardware design, the exoskeleton hardware module adopts a detachable modular structure, making the device more flexible and user-friendly for patients. Weighing less than 400 g, the joint actuation utilizes compact electric linear actuators, ensuring adequate mechanical support without restricting natural hand movement [9, 10]. The sensor module integrates a three-axis inertial measurement unit (IMU) and surface electromyography (sEMG) sensors to synchronously capture angular velocity and posture information of fingers and wrist, as well as activation intensity of muscle groups (such as flexor digitorum superficialis and extensor digitorum), enabling accurate determination of patient motor intent [15, 16].

To provide an immersive training experience, the virtual reality environment was developed based on the Unity engine, supporting multiple task scenarios (such as virtual block stacking and finger tapping rhythm tasks) [11, 13]. Through mechanical simulation and gamification feedback mechanisms, the system effectively enhances patient engagement and training adherence. The cloud AI engine is responsible for fused assessment of multimodal inputs, utilizing a hybrid model combining random forest and support vector regression (SVR) for data analysis [24, 27], ensuring precise rehabilitation progress assessment and training parameter adjustment. The engine is deployed on AWS edge computing nodes, supporting bidirectional communication with local controllers via MQTT message queues, ensuring real-time performance and response speed [30].

**Figure 2:**
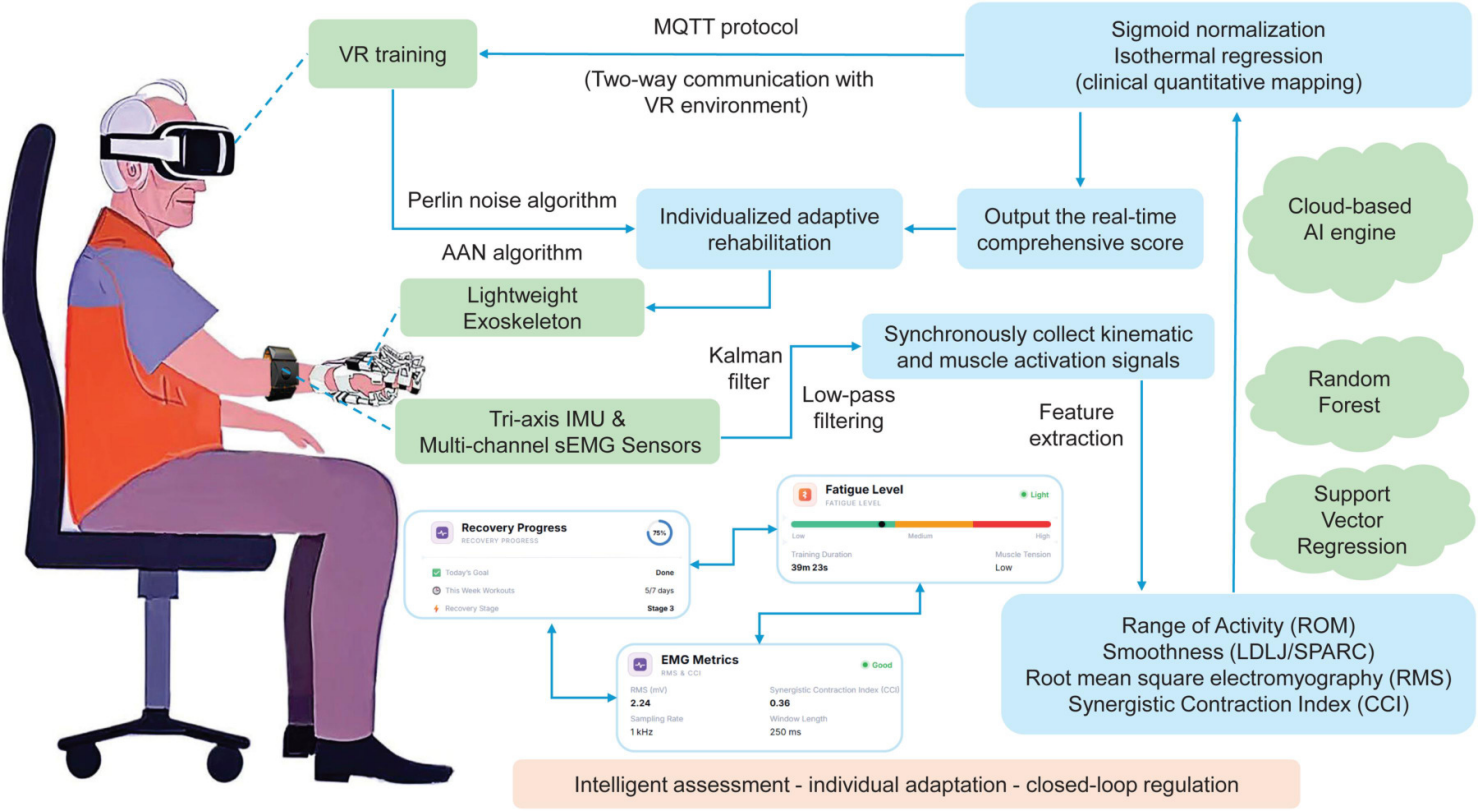
Overall operational framework. The system workflow illustrates the complete perception-decision-action cycle: from sensor data acquisition through preprocessing, feature extraction, AI-based scoring, to adaptive control adjustments of both exoskeleton assistance and VR task parameters.

The entire system’s multi-threaded parallel data processing is realized under the RT-Thread embedded operating system, ensuring real-time data processing and feedback, with end-to-end communication latency controlled within 50 ms, enabling the perception-feedback loop to take effect in real time. The overall system operation framework is shown in Figure 2, while Table 1 lists the key parameters of each system module, demonstrating the performance indicators and design requirements of hardware and software modules.

**Table 1:** System parameters for rehabilitation system.

Module	Parameter	Description	Value
Exoskeleton Hardware	Weight	Total weight	≤400 g
	Drive Mechanism	Joint actuation type	High-torque servo motors (PWM)
	Degree of Freedom	Per finger	3 DoF (per finger)
	Max Torque	Per joint	2 Nm (per joint)
	Max Speed	Movement speed	$300^{\circ }$/s
Sensor Module	IMU	Movement tracking	3-axis accel. + gyro.
	sEMG Sensor	Muscle activation	Surface EMG
	Sampling Freq.	Data sampling rate	IMU: 100 Hz; sEMG: 1,000 Hz
VR Interaction	Platform	Development platform	Unity 3D Engine
	Task Types	Virtual tasks	Block stacking, tapping, gripping
	Interface	User interface	Gamified with real-time feedback
Cloud AI Engine	Data Preprocessing	Processing algorithm	Butterworth low-pass filter
	Feature Extraction	Extracted features	Jerk, ROM, muscle activation
	ML Model	Scoring model	Random Forest + SVR
	Rehabilitation Score	Progress evaluation	Composite score (0–1)
Adaptive Control	Feedback Algorithm	Adaptive assistance	AAN based on rehab score
	Control Delay	Max processing delay	≤50 ms
Communication	Protocol	Communication type	MQTT Protocol
	Connection Type	Connection method	Wireless (Wi-Fi/BLE)
System Battery	Battery Type	Power source	Lithium-ion
	Battery Life	Operation time	≥6 h

### 3.2 Hardware design

#### 3.2.1 Exoskeleton mechanical structure

**Figure 3:**
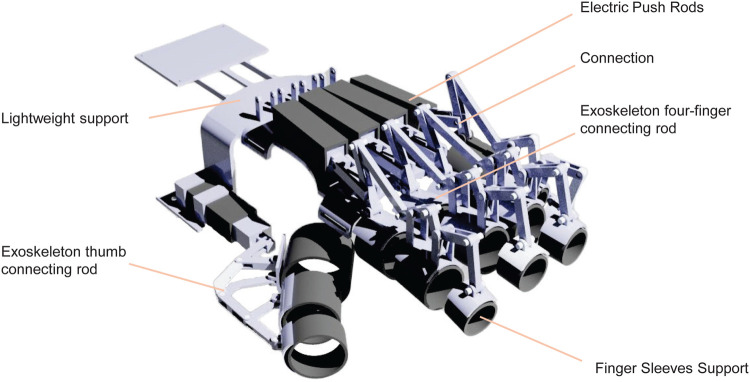
Exoskeleton structure.

The hand exoskeleton (see Figure 3) employs a linkage mechanism to drive each joint, thereby achieving effective restoration of hand function. Through precise mechanical design, the linkage mechanism enables the exoskeleton to mimic natural hand movement patterns, providing patients with efficient passive and active assistive motion. The total weight of the entire exoskeleton is controlled below 400 g, ensuring that patients maintain high comfort during prolonged wearing without excessive pressure on the skin. Specifically, the stress on the skin remains below 0.5 N/cm^2^, and the thermal comfort index (THI) is maintained below 26^∘^C, ensuring that even during extended use, patients’ skin does not experience adverse reactions due to overheating or discomfort.

In terms of the power system, the exoskeleton’s actuation consists of five electric linear actuators, each with a response time of less than 100 ms and a precision of $\pm 0.1^{\circ }$, enabling precise control of each joint’s movement. The torque design of the electric actuators ensures they can effectively bear loads exceeding 20 N, sufficient to handle common grasping tasks in daily life, such as gripping bottles and keys. Thus, patients can not only perform fine grasping movements but also execute some routine daily tasks, enhancing independence in daily living.

#### 3.2.2 Exoskeleton electronic control system

**Figure 4:**
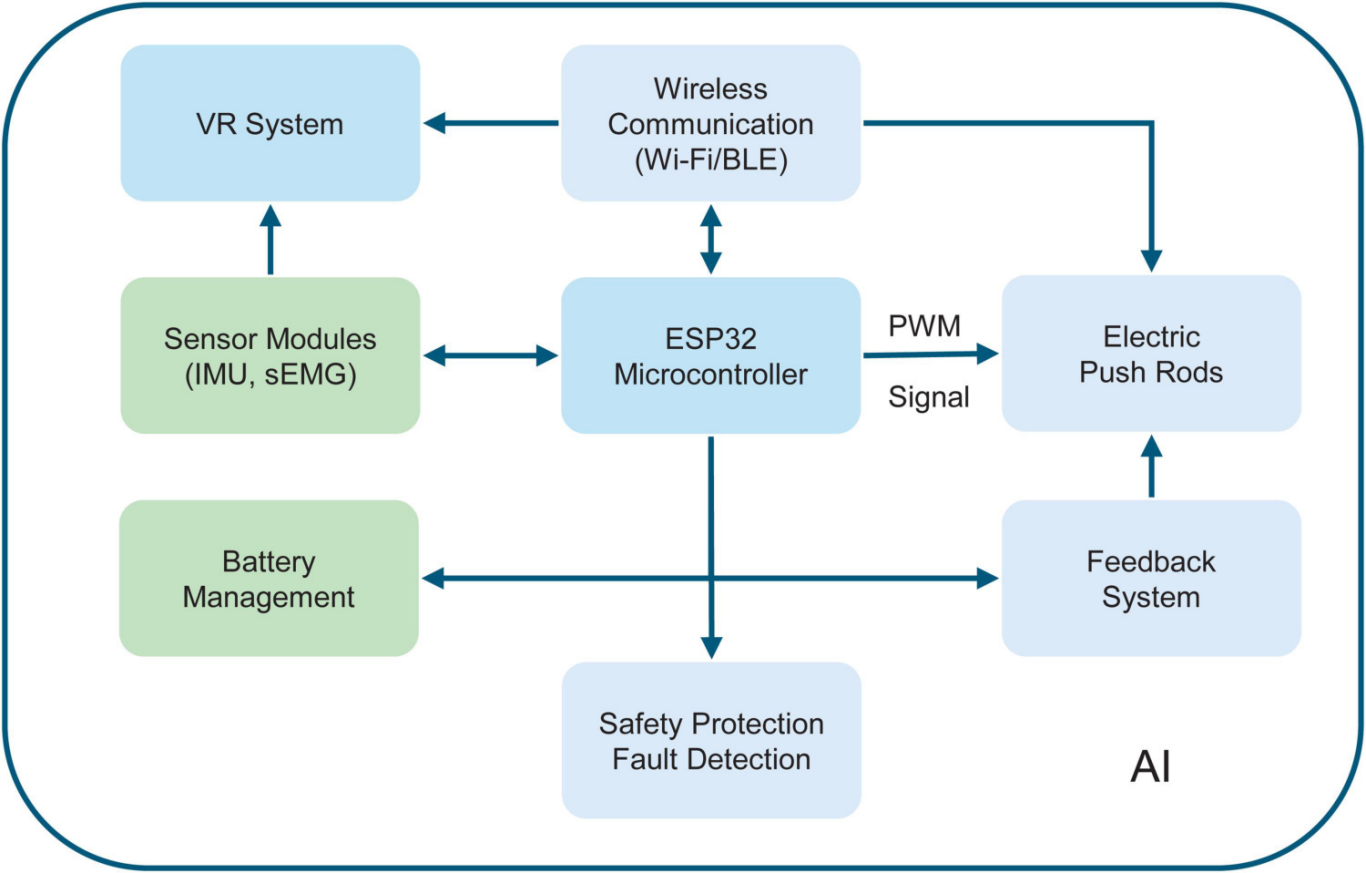
Electronic control system of the exoskeleton.

The core control unit of the system (see Figure 4) is dominated by an ESP32 microcontroller, which adopts a dual-core Xtensa LX6 architecture with a main frequency of 240 MHz and supports WiFi and BLE wireless communication. It processes motion and physiological data collected from exoskeleton sensors (such as IMU and sEMG sensors) in real time, thereby identifying patients’ movement intentions and generating corresponding control commands. Five electric linear actuators serve as the power source of the exoskeleton, capable of receiving commands from the control unit through PWM signals to drive the movement of hand joints. This meets the demands of daily tasks such as gripping bottles or keys.

The system’s feedback mechanism corrects commands through actual joint movement data collected by sensors, ensuring the accuracy and smoothness of each movement, enhancing patients’ motor recovery effects. Through WiFi and BLE functions, the exoskeleton can wirelessly transmit data to smart devices (such as smartphones and computers), supporting remote adjustment of treatment plans and real-time monitoring of rehabilitation progress by doctors or caregivers.

To ensure safety during use, the electronic control system also incorporates multiple safety protection mechanisms, including overload protection, short-circuit protection, and temperature monitoring, ensuring stable operation of the device. The system also conducts continuous self-checks, promptly detecting potential faults and issuing alarms to ensure patient safety and long-term stability of the device.

#### 3.2.3 Data acquisition and processing

The system acquires joint motion and muscle electrical activity data in real time through inertial measurement units (IMU) and surface electromyography (sEMG) sensors. The IMU sensor measures the acceleration and angular velocity of the hand to obtain the motion state of joints. The IMU typically consists of a three-axis accelerometer and a three-axis gyroscope, measuring linear acceleration and rotational angular velocity respectively. During joint movement, the IMU calculates angular changes of finger joints based on sensor output data (see Equation 1):

θ(t)=∫ω(t)dt (1)

where θ(t) is the angular change of the joint, ω(t) is the angular velocity measured by the gyroscope, and the integration operation converts it to angular information.

The accelerometer of the IMU is used to obtain the linear acceleration of joints (see Equation 2):

Position(t)=∫a(t)dt (2)

where Position(t) represents the positional change of the joint, and a(t) is the acceleration signal obtained through the accelerometer.

The sEMG sensor is used to detect muscle electrical activity, reflecting muscle activation. By acquiring electromyographic signals, the system can determine the patient’s intention and thus judge whether assistive movements are needed. Electromyographic signals are typically evaluated using time-domain features to assess the degree of muscle activation (see Equation 3):

$$\text{RMS}=\sqrt{\frac{1}{N}\sum_{i=1}^{N}x_{i}^{2}}$$ (3)

where $x_{i}$ is the sampled value of the electromyographic signal, and N is the number of sampling points.

The raw data collected by sensors are often affected by noise and interference, so filtering processing is required. In this system, a low-pass filter is used to remove high-frequency noise and extract useful motion signals (see Equation 4):

$$H(s)=\frac{1}{1+{\left(\frac{s}{\omega_{c}}\right)}^{2n}}$$ (4)

where $\omega_{c}$ is the cutoff frequency of the filter, n is the order of the filter, and s is the complex frequency domain variable. By adjusting the cutoff frequency and order, appropriate filtering effects can be selected to remove high-frequency noise while retaining low-frequency joint motion signals.

After acquiring and preprocessing IMU and sEMG data, data fusion and motion intention recognition are performed next. The purpose of data fusion is to synthesize signals from different sensors into a more accurate set of control data. In this system, the Kalman filtering algorithm is employed to fuse IMU data and reduce errors between accelerometer and gyroscope data (see Equation 5):

$${\hat{x}}_{k|k}={\hat{x}}_{k|k-1}+K_{k}(z_{k}-H_{k}{\hat{x}}_{k|k-1})$$ (5)

where ${\hat{x}}_{k|k}$ is the estimated value at the current moment, $K_{k}$ is the Kalman gain, $z_{k}$ is the actual measured value from the sensor, $H_{k}$ is the observation matrix, and $P_{k|k}$ is the error covariance matrix. Kalman filtering can effectively fuse data from different sensors to provide more accurate angle estimates.

Through analysis of sEMG signals, the patient’s intention to perform a certain action can be further determined. Support vector machines (SVM) are used to classify sEMG signals and identify patient intentions (see Equation 6). The trained model can classify input electromyographic signals in real time and generate corresponding motion commands:

$$f(x)=w^{T}x+b$$ (6)

where x is the input sEMG feature vector, w is the model’s weight vector, and b is the bias term. Through training, the model automatically adjusts weights and bias to optimize classification performance.

After data acquisition, preprocessing, filtering, and fusion, the system finally generates motion commands and transmits them to electric linear actuators via PWM signals. These commands control joint movements and are corrected through real-time feedback mechanisms to ensure the accuracy and smoothness of movements. All acquisition, processing, and output processes are performed in real time to ensure timely system response.

### 3.3 Virtual reality environment

#### 3.3.1 Environment construction and task modules

**Figure 5:**
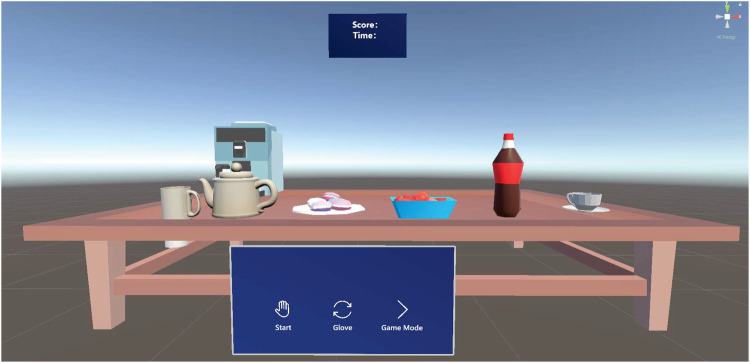
Immersive VR rehabilitation environment and gamified task modules.

In the exoskeleton system, the construction of a virtual reality (VR) environment provides patients with an immersive 3D rehabilitation training scenario (see Figure 5) [11, 13]. This scenario simulates a typical daily living environment, centered on a table with some common objects placed on it. The patient’s task is to grasp these objects through the exoskeleton system for rehabilitation training. The volume of the virtual environment is approximately 2 m × 2 m × 2 m, with lighting set to a soft 500–1,000 lux to reduce the occurrence of visually induced motion sickness (VIMS), ensuring its incidence rate is below 10%. This environment not only provides realistic physical interaction but is also equipped with a scoreboard that displays the patient’s score in real time, enhancing the fun and challenge of training [17].

The core task modules of this virtual environment (gamification task modules) include:

##### 3.3.1.1 Object grasping task

On the virtual desktop, patients need to grasp and place 5–10 different daily objects (such as bottles, cups, etc.). Each object has a size of 10–50 cm^3^ and is rendered with realistic physical reactions through PhysX simulation of gravity (g=9.81 m/s^2^). Patients need to grasp and place as many items as possible within a specified time to train grasping, releasing, and finger dexterity.

##### 3.3.1.2 Finger tapping task

This task simulates keyboard input operations in activities of daily living (ADL). Patients need to complete finger tapping movements according to the rhythm displayed on the screen. The task controls the rhythm through a Metronome component with a frequency of 1–3 Hz, aiming to improve patients’ finger coordination, fine motor skills, and reaction speed.

##### 3.3.1.3 Object grasping and releasing cycle

In this task, patients need to continuously grasp and release virtual objects, specifically training thumb opposition function. The task sets a grip force threshold greater than 2 N, ensuring that patients can enhance finger grip strength and dexterity during training.

These task modules follow the progressive overload principle [8], starting from simple single degree-of-freedom (1 DoF) tasks and gradually increasing task difficulty and degrees of freedom, ultimately transitioning to multi-degree-of-freedom (4 DoF coordination) tasks to improve patients’ hand function. Each training session has a total duration of 20 min, and through dynamic adjustment of task difficulty, ensures that patients always maintain an appropriate load range during training to promote efficient rehabilitation progress. Real-time score feedback on the scoreboard further motivates patients to actively participate in training and maintain high levels of engagement [12].

#### 3.3.2 Interaction and adaptive mechanisms

**Figure 6:**
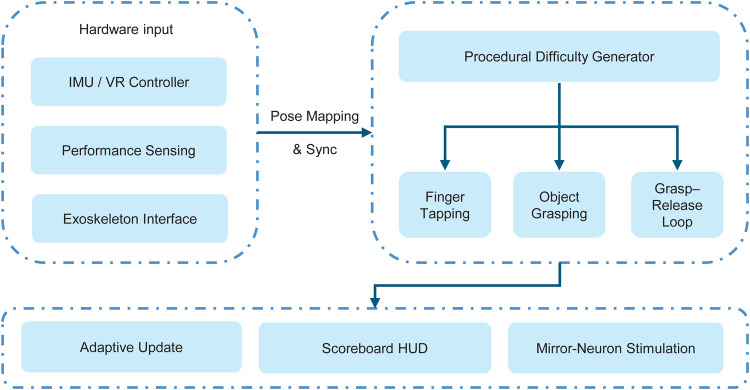
Interaction and adaptation pipeline for VR hand-exoskeleton rehab.

To achieve a more immersive and personalized rehabilitation experience, this system designed multi-level interaction and adaptive mechanisms in the VR environment (see Figure 6) [23]. This mechanism combines motion mapping, difficulty adjustment, and neural feedback, maintaining balance between physiological load and cognitive challenge through real-time data perception and dynamic task generation.

In terms of task adaptive design, the system employs procedural generation algorithms to achieve dynamic construction of task scenarios. Training difficulty is adjusted in real time according to the user’s range of motion (ROM): when baseline ROM is detected to be below $45^{\circ }\pm 3^{\circ }$, target object size is automatically enlarged by 20% to reduce operational difficulty; meanwhile, Perlin noise functions [28] are used to generate randomized object layouts, avoiding monotony caused by repetitive training. Path planning is based on Unity’s built-in NavMesh system and A* algorithm with a step size of 0.1 m and heuristic function h(n) as Euclidean distance, thus ensuring smoothness and predictability of virtual target movement.

In terms of motion interaction mapping, users input posture through VR controllers or IMU sensors, with signals transmitted via Bluetooth Low Energy (BLE 2.4 GHz) to the exoskeleton control unit, with communication delay controlled within 20 ms [30]. Data transmission adopts a packet protocol with a maximum transmission unit (MTU) of 512 B, representing spatial posture with quaternions $\left(q_{w},q_{x},q_{y},q_{z}\right)$ with precision up to 0.001. The exoskeleton responds in real time to motion changes in the virtual scene, achieving “virtual-real synchronization” operational experience.

To further stimulate the rehabilitation participant’s training initiative and neuroplasticity [4], the system introduces a mirror neuron stimulation mechanism. When users complete effective grasping actions, the system triggers visual and auditory rewards (such as particle effects and pleasant sounds), forming a positive reinforcement experience similar to “dopamine feedback”; when actions fail, gradient highlighting and vibration feedback are used for prompts, with vibration intensity ranging from 0%–100%, guiding users to gradually adjust hand posture. This positive-negative feedback combination mechanism can enhance the brain’s consolidation and relearning effects on movement patterns [31].

### 3.4 AI engine

#### 3.4.1 Data preprocessing and feature extraction

To support millisecond-level closed-loop control of assistance-as-needed (AAN), the AI engine employs a streaming pipeline of “calibration-cleaning-alignment-segmentation-featurization-standardization/dimensionality reduction.” The overall system operates in dual channels: the online channel uses short windows of 128–256 ms (50% overlap) for incremental updates, driving real-time scoring and parameter adaptation; the offline channel performs summary assessment and treatment trend analysis based on long windows of 5–30 s or entire task segments.

In terms of time synchronization, both ESP32 (exoskeleton/sensors) and Unity (VR) are calibrated to UTC time base via NTP, with the cloud side performing delay estimation and compensation based on arrival time. sEMG (1 kHz), IMU (100–200 Hz), and interaction logs (20–60 Hz) are resampled to a unified 100 Hz common timeline, maintaining amplitude consistency through linear interpolation or zero-order hold methods. Cross-modal delay is estimated through cross-correlation maximum value to determine propagation lag $\Delta t^{*}$, used for phase alignment of fast channels (sEMG) (see Equation 7):

$$\Delta t^{*}=\arg\max_{\tau }\;\mathrm{xcorr}\left(e(t),\;\dot{\theta }(t+\tau )\right)$$ (7)

Sensor calibration estimates IMU zero bias and scale factors to obtain corrected angular velocity $\tilde{\omega }$ and acceleration $\tilde{a}$; attitude estimation employs complementary or extended Kalman filtering (EKF) to fuse gyroscope and accelerometer data, using quaternions $q=[q_{w},q_{x},q_{y},q_{z}]$ for real-time updates, separating gravity when needed to obtain pure motion components. Subsequently, by guiding subjects through active/passive limits, individualized baseline range of motion ${\text{ROM}}_{\text{base}}$ is calibrated for normalization purposes.

In terms of noise removal and artifact suppression, sEMG employs 4th-order bandpass filtering at 20–450 Hz and notch filtering at 50/60 Hz, followed by rectification and 3–10 Hz low-pass filtering to obtain envelope e(t). IMU signals use 2–4th order low-pass filtering at 5–10 Hz to remove high-frequency jitter, with mild detrending applied to angular velocity. Outliers are removed through windowed Hampel/MAD methods, with short gaps filled by neighborhood interpolation.

Segment division uses sliding windows of 128–256 ms with step sizes of 64–128 ms for online inference; offline assessment is performed on entire task segments. Note that “task segment” refers to a complete functional trial (e.g., one reach-grasp-release cycle lasting 5–30 s), while “sliding window” refers to the 200 ms computational units extracted within each task segment for real-time feature computation. During each 20-min session, participants completed approximately 15–20 task trials, yielding a total of 8,946 annotated task segments across all 498 sessions. Within each task segment, overlapping 200 ms sliding windows were extracted for real-time scoring and control. For intention and event detection, the Teager-Kaiser energy operator (TKEO) enhances energy bursts combined with adaptive thresholding to identify active intention (see Equation 8):

$$\Psi [x(n)]=x(n{)}^{2}-x(n-1)\;x(n+1)$$ (8)

When consecutive sampling points exceed the threshold, motion onset is determined; events such as task start, grasp, release, and completion are precisely delimited by VR logs, used to align homogeneous trials and calculate task-level metrics.

Feature extraction follows a hierarchical online/offline design to balance real-time performance and discriminative power. In the kinematic and dynamic dimension, range of motion is defined as (see Equation 9):

$$\text{ROM}=\theta_{\max}-\theta_{\min},\;{\text{ROM}}_{n}=\frac{\text{ROM}}{{\text{ROM}}_{\text{base}}}$$ (9)

Velocity and acceleration statistics (such as $\bar{v},v_{\text{peak}}\;,\sigma_{v}$, and time to peak velocity TTPV) are extracted, along with smoothness metrics including the dimensionless jerk metric LDLJ (see Equation 10):

$$\text{LDLJ}=\log\left(\frac{T^{5}}{A^{2}}\int_{0}^{T}\|\dddot{x^{...}}(t){\|}^{2}\;dt\right)$$ (10)

The spectral arc length (SPARC) smoothness, path efficiency $\eta =L_{\text{straight}}/L_{\text{actual}}$, tremor energy ratio at 4–12 Hz, target deviation RMSE, endpoint error, and dwell ratio are also calculated. If joint torque τ is available, assistive work is measured by energy integral (see Equation 11):

$$E_{\text{assist}}=\int \tau (t)\;\dot{\theta }(t)\;dt$$ (11)

In the electromyographic dimension, time-domain features include RMS, mean absolute value (MAV), waveform length (WL), zero crossing count (ZC), slope sign changes (SSC), and integrated EMG (IEMG). Frequency-domain and fatigue features are characterized by mean frequency and median frequency (see Equation 12):

$$\text{MNF}=\frac{\sum f\;P(f)}{\sum P(f)}$$ (12)

Spectral centroid, bandwidth, and their temporal slopes are calculated to characterize fatigue trends. To quantify antagonist muscle co-contraction, the co-contraction index (CCI) is used (see Equation 13):

$$\text{CCI}=\frac{\sum_{t}\min\left(e_{a}(t),e_{b}(t)\right)}{\sum_{t}\max\left(e_{a}(t),e_{b}(t)\right)}$$ (13)

Electromechanical delay (EMD) between EMG onset and motion onset is estimated to reflect neural drive to mechanical response coupling efficiency. When necessary, non-negative matrix factorization (NMF) can extract muscle synergy basis vectors and activation time series in offline analysis.

Cross-modal coupling features measure consistency between EMG and motion through windowed cross-correlation peaks and lags, as well as mutual information (see Equation 14):

$$I(e;\dot{\theta })$$ (14)

Additionally, the lag Δt of sEMG onset relative to velocity onset characterizes intention consistency, and the “effort-performance ratio” relates success rate per unit or ROM per unit to amplitude-related metrics like IEMG and RMS to assess efficiency and cost. Task/behavioral level metrics from VR logs include success rate, completion time, error rate, inter-trial coefficient of variation, etc., and calculate difficulty index and throughput based on Fitts’ law (see Equation 15):

$$\text{ID}=\log_{2}\left(\frac{D}{W}+1\right),\;\text{TP}=\frac{\text{ID}}{T}$$ (15)

To reduce inter-subject variability and improve longitudinal comparability, all features are standardized using individualized baselines. Common methods include z-score normalization using mean and standard deviation from calibration period, or robust scaling using median and IQR; amplitude-related features (such as ROM, IEMG) use ratio normalization relative to baseline (referenced to the mean of initial three treatment sessions). To suppress slow drift, exponential moving averages are used for adaptive baseline updating (see Equation 16):

$$\mu_{t}=\alpha x_{t}+(1-\alpha )\mu_{t-1}\;(\alpha \approx 0.01-0.05)$$ (16)

Task condition matching is performed through VR metadata, comparing under the same difficulty or target distance conditions to avoid confounding.

Feature selection and dimensionality reduction follow the principle of “lightweight online, rich offline.” The online phase combines information gain/mutual information and variance filtering to remove low-variance and high-redundancy features, controlling online computed features to approximately 20–40 to meet a computational budget of less than 5 ms per window; the offline phase uses mRMR for pre-screening, then employs PCA or factor analysis to retain no less than 95% cumulative variance, used to construct comprehensive indices such as “smoothness composite score” and “fatigue composite score.”

In terms of computational resources and robustness, edge nodes maintain median time consumption for single-window preprocessing and featurization not exceeding 5 ms under conditions of 100 Hz input and 16-channel sEMG. Facing short gaps of no more than 10%, the system maintains robustness through feature subset voting and masking mechanisms; when data quality score Q falls below threshold τ, real-time scoring is downweighted and recalibration prompts are triggered. All features record metadata such as filter order, window length, and thresholds, ensuring traceability for clinical review and experimental reproduction.

#### 3.4.2 Scoring model training and online inference

This section aims to map the multimodal features obtained in Section 2.4.1 to a clinically consistent, real-time updatable comprehensive rehabilitation score $S_{t}\in [0,1]$, which is used both for longitudinal quantification of progress and as a control variable for assistance-as-needed (AAN). The training phase adopts a strategy combining hierarchical supervised learning and calibration: using multi-task objectives composed of clinical scales and task-level objective metrics as supervisory signals, training base learners and fusers under a subject-grouped cross-validation framework; the online phase performs lightweight inference and smoothing on the edge side, driving exoskeleton assistance and VR difficulty adaptation with robust closed-loop control laws.

Training data consists of $D=\{(x_{i},y_{i}){\}}_{i=1}^{N}$, where $x_{i}$ is the feature vector of the ith window or trial, and $y_{i}=[y_{i1},\ldots ,y_{iM}]$ is multi-task labels (such as standardized FMA-UE, ARAT, ${\mathrm{ROM}}_{n}$, smoothness metrics, etc.). The selection of random forest (RF) and support vector regression (SVR) as base learners is motivated by three practical considerations: (1) computational efficiency for real-time deployment—RF achieves sub-5 ms inference time on embedded hardware (ESP32 dual-core 240 MHz) with model sizes under 2 MB, whereas deep learning architectures (LSTM, Transformer) require GPU acceleration and incur 50–200 ms latency incompatible with closed-loop control; (2) robustness to small-sample scenarios—this study’s dataset of 24 subjects and 498 sessions is insufficient for training deep models without severe overfitting, while RF and SVR excel in low-data regimes through ensemble averaging and kernel-based generalization; (3) interpretability for clinical validation—RF feature importance and SVR support vectors enable transparent inspection of decision mechanisms, facilitating clinician trust and regulatory approval, whereas black-box neural networks obscure such insights. Hyperparameters were optimized through 5-fold GroupKFold cross-validation: RF employs 200 trees with maximum depth 8, minimum samples per leaf 5, and Gini impurity criterion; SVR uses radial basis kernel with γ=0.01, regularization C=10, and ϵ=0.1. Ridge regression meta-learner applies $L_{2}$ regularization λ=0.5. Base learners select random forest regressors and radial basis kernel SVR to fit each task objective, obtaining ${\hat{y}}_{i}^{\left(\text{RF}\right)}$ and ${\hat{y}}_{i}^{\left(\text{SVR}\right)}$. Subsequently, a stacked fusion approach trains a linear ridge regression meta-learner g(⋅), with input as concatenated vectors of both base learner predictions and a few contextual elements (such as current task difficulty, individual ROM baseline), outputting fused predictions for each task ${\hat{y}}_{i}=g([{\hat{y}}_{i}^{\left(\text{RF}\right)},{\hat{y}}_{i}^{\left(\text{SVR}\right)},c_{i}])$.

To improve robustness to heteroscedasticity and outliers, training minimizes the weighted Huber loss sum with task weights, and standardizes scale differences across different tasks. The loss function is (see Equation 17):

$$L=\sum_{i=1}^{N}\sum_{m=1}^{M}w_{m}\;H_{\delta }\left(y_{im}-{\hat{y}}_{im}\right)$$ (17)

where $H_{\delta }(r)=\frac{1}{2}r^{2}$ when |r|≤δ, otherwise $H_{\delta }(r)=\delta (|r|-\frac{1}{2}\delta )$, and $w_{m}$ is set by clinical relevance or expert priors and fine-tuned on validation sets. To avoid subject leakage, GroupKFold (grouped by subjects) cross-validation is used to estimate generalization performance and select hyperparameters, with early stopping and feature sparsity regularization to suppress overfitting.

The construction of the FMA-UE proxy for each 200 ms window addresses the temporal resolution mismatch between real-time scoring (window-level) and clinical assessments (session-level measured only at baseline and week 4). Proxy labels $y_{\text{FMA-proxy}}$ were generated through three complementary strategies: (1) Linear interpolation—for each patient, FMA-UE scores at baseline (t=0) and week 4 (t=T) were linearly interpolated across intermediate sessions ($t_{s}$) assuming monotonic improvement: ${\text{FMA}}_{s}={\text{FMA}}_{0}+\frac{t_{s}}{T}({\text{FMA}}_{T}-{\text{FMA}}_{0})$, then distributed uniformly across windows within each session; (2) Biomechanical anchoring—within-session window-level proxies were refined by normalizing multimodal features (ROM, SPARC, RMS) relative to session-level statistics and scaling by the interpolated session FMA, creating proxies that reflect intra-session variability while maintaining inter-session monotonicity; (3) Expert annotation—a subset of 50 video-recorded training trials were independently scored by two blinded occupational therapists (OT1, OT2) using a simplified 0–10 analog scale correlated with FMA-UE items, achieving inter-rater reliability Cohen’s weighted κ=0.78 (95% CI [0.71, 0.85]), with AI proxy scores showing Spearman ρ=0.72 (p<0.001) against therapist consensus. Ground-truth labels for offline training thus combined interpolated clinical anchors (80% of data), biomechanical-normalized proxies (15%), and expert-annotated samples (5%) via weighted ensemble to balance temporal coverage and measurement fidelity. Leave-one-subject-out cross-validation (LOSOCV) was conducted to assess generalization to new patients: the model was trained on 23 subjects and tested on the held-out subject, repeated 24 times, yielding mean $R^{2}=0.68\pm 0.09$ (range 0.51–0.79) and MAE =3.8±0.7 points, confirming adequate generalization despite inter-subject heterogeneity. Test-retest reliability of online scores was evaluated on 8 patients undergoing duplicate sessions 48 h apart, achieving ICC(2,1) = 0.84 (95% CI [0.76, 0.91]), indicating excellent consistency.

Construction of the comprehensive score proceeds in two steps. First, fused predictions are standardized, making each task’s dimensionless component $z_{im}=\text{zscore}({\hat{y}}_{im})$ or robust scaling relative to baseline. Then, through a monotonic interpretable linear-sigmoid mapping, interval 0–1 comprehensive values are obtained, where task coefficients $\alpha_{m}\geq 0$ and $\sum_{m}\alpha_{m}=1$, (a,b) are calibration parameters (see Equation 18):

$$S_{i}=\sigma \left(a\;\sum_{m=1}^{M}\alpha_{m}z_{im}+b\right),\;\sigma (u)=\frac{1}{1+e^{-u}}$$ (18)

To align with the individualized functional recovery “baseline-healthy reference” framework, the system simultaneously provides a reference mapping for interpretation and review (see Equation 19). Given the individual calibration period expectation $\mu_{0}$ and healthy control expectation $\mu_{\text{ref}}$ (see Equation 19):

$$S_{i}^{\text{ref}}=\text{clip}\left(\frac{{\hat{y}}_{i}-\mu_{0}}{\mu_{\text{ref}}-\mu_{0}},0,1\right)$$ (19)

Through isotonic regression or Platt scaling, $S_{i}^{\text{ref}}$ and $S_{i}$ are aligned on the validation set to obtain final scores that are both smooth and clinically monotonic. Model calibration comprehensively optimizes Spearman rank correlation, $R^{2}$, MAE, and test-retest consistency (ICC), where ICC estimation uses two-way random absolute agreement type ICC(2,1) to ensure longitudinal comparability.

Online inference adopts a “feature stream → dual base learners → fusion → calibration → smoothing” pipeline-style recursion. Instantaneous scores obtained for each sliding window are denoted as $S_{t}$, then exponentially smoothed to suppress instantaneous jitter and form control-friendly state variables (see Equation 20):

$${\tilde{S}}_{t}=\lambda S_{t}+(1-\lambda ){\tilde{S}}_{t-1},\;\lambda \in (0,1)$$ (20)

Meanwhile, uncertainty estimation $u_{t}$ is constructed using random forest out-of-bag variance and SVR residual history; when $u_{t}$ is high, control gain is reduced and data recalibration prompts are triggered. To resist concept drift, the system performs lightweight domain adaptation at the session level, updating only fusion layer bias terms and a few scaling parameters to align session mean with baseline. Given a session’s target exponential moving average as $\bar{S}$ and predicted mean as $\bar{\hat{S}}$, online bias correction is (see Equation 21):

$$b_{t+1}=b_{t}+\eta (\bar{S}-\bar{\hat{S}}),\;\eta \in (0,1)$$ (21)

The AAN control law takes smoothed score ${\tilde{S}}_{t}$ as input, continuously adjusting exoskeleton assistance and VR difficulty, avoiding “takeover” and oscillation through dead zones and rate limits (see Equation 22). Given normalized assistive force or torque $u_{t}\in [u_{\min},u_{\max}]$, target score $S^{*}$ (e.g., set at 0.7–0.8 according to target challenge level):

$$u_{t}=\text{clip}\left(u_{t-1}+k_{u}(S^{*}-{\tilde{S}}_{t}),u_{\min},u_{\max}\right)$$ (22)

The AAN control law constitutes the core adaptive mechanism of this system, designed to dynamically balance “challenge” and “capability” during rehabilitation training, ensuring patients remain within Vygotsky’s zone of proximal development—neither falling into passive dependency due to excessive assistance nor experiencing frustration from overly difficult tasks. Compared to traditional fixed-assistance modes in rehabilitation exoskeletons or instant adjustment methods based on local motion signals, this AAN control law innovatively uses the AI composite score ${\tilde{S}}_{t}$ as the sole control input to achieve cross-trial, cross-session task-level adaptation, synchronously regulating both physical assistance (exoskeleton torque) and cognitive load (VR task difficulty) to form a “dual-axis synergistic” personalized rehabilitation closed loop.

The control gain $k_{u}>0$ is set to 0.05 (optimized through offline simulation and pilot experiments with 3 healthy subjects), and ${\tilde{S}}_{t}$ is the smoothed score from the tth control cycle (200 ms sliding window). To prevent control signal chattering and oscillation, three constraint mechanisms are imposed: (1) Rate limiting: constraining single-step adjustment magnitude $|u_{t}-u_{t-1}|\leq r_{\max}$ (set $r_{\max}=0.02$, i.e., maximum 2% change per cycle) to avoid sudden assistance force changes that may startle or destabilize patients; (2) Dead zone: freezing updates when $|S^{*}-{\tilde{S}}_{t}|<\epsilon$ (set ϵ=0.05) to avoid frequent adjustments due to minor fluctuations within the target range; (3) Hysteresis band: setting upper and lower thresholds $\left[\theta_{\text{low}},\theta_{\text{high}}]=[0.65,0.85\right]$, triggering assistance direction switching (increase or decrease) only when ${\tilde{S}}_{t}$ crosses thresholds, further suppressing jitter.

Synchronized with exoskeleton assistance, the second channel regulates VR task difficulty parameter $d_{t}$, encompassing multi-dimensional attributes of virtual targets including size (diameter range 5–15 cm), movement speed (0.1–0.5 m/s), reachable distance (20–50 cm), and Fitts difficulty index $\text{ID}=\log_{2}(D/W+1)$. The adjustment follows a mapping isomorphic to $u_{t}$ (see Equation 23):

$$d_{t}=\text{clip}\left(d_{t-1}+k_{d}({\tilde{S}}_{t}-S^{*}),d_{\min},d_{\max}\right)$$ (23)

where $k_{d}<0$ (set to −0.08) indicates inverse regulation between difficulty and score—when ${\tilde{S}}_{t}$ increases (excellent patient performance), task difficulty rises (smaller target size, higher speed); conversely, difficulty decreases to avoid frustration. Similar rate limiting, dead zone, and hysteresis processing ensure smooth VR scene transitions and continuous user experience. The coupling effect of dual-channel control is: when patient rehabilitation progresses significantly (${\tilde{S}}_{t}↑$), the system reduces exoskeleton assistance ($u_{t}↓$) while synchronously increasing VR challenge ($d_{t}↑$), compelling patients to engage actively and strengthening neuromuscular activation; when patients experience fatigue or performance decline (${\tilde{S}}_{t}↓$), assistance increases ($u_{t}↑$) and tasks simplify ($d_{t}↓$), maintaining training sustainability and preventing secondary injury.

To ensure clinical safety, the AAN control law integrates multi-level protection mechanisms: (a) Score lower-bound protection: when ${\tilde{S}}_{t}<\theta_{\text{safe}}$ (set to 0.4, indicating severe performance degradation or sensor fault), the system forcibly enters protection mode, elevating $u_{t}$ to $u_{\max}$ (maximum assistance) and freezing upward adjustment of $d_{t}$, while triggering auditory alerts for therapist intervention; (b) Uncertainty protection: utilizing uncertainty estimation $\sigma_{t}$ constructed from random forest out-of-bag variance and SVR residuals, when $\sigma_{t}>\sigma_{\text{thresh}}$ (threshold set to 0.15), control gain is reduced ($k_{u}\leftarrow 0.5k_{u}$) and data recalibration is prompted, avoiding aggressive adjustments when model predictions are unreliable; (c) Physiological signal monitoring: if sEMG remains below 10% of baseline and IMU detects stationary state (velocity $<2^{\circ }$/s) for over 5 s, the system infers patient withdrawal or attention loss, pauses the task, and logs the event for post-hoc analysis.

Compared to the variable admittance control (V-AC) in reference [20] and resist-as-needed (RAN) control in reference [21], this AAN control law achieves paradigm breakthroughs across four dimensions: control hierarchy, input signals, closed-loop scope, and rehabilitation objectives. (1) Control hierarchy: V-AC [20] employs an admittance model adjusting damping B and inertia M parameters in real time to track user motion intent, essentially low-level motion control responding instantaneously (millisecond scale) to force/velocity signals; RAN [21] uses a PD controller with online impedance adaptation, likewise local parameter optimization focused on quantifying fatigue-induced mechanical impedance changes. In contrast, this AAN control law uses AI composite score ${\tilde{S}}_{t}$ (integrating 18 kinematic features, 12 sEMG features, and 3 task performance metrics) as sole input, performing adaptive regulation at task-level/session-level (minute to week time scales), belonging to high-level rehabilitation strategy control that shapes overall rehabilitation trajectory rather than optimizing individual actions. (2) Input signals and information richness: references [20, 21] rely on single-modality signals (force sensors, angular velocity, or position/velocity errors) that only reflect instantaneous motion states or trajectory tracking accuracy, lacking perception of deeper physiological states (muscle activation patterns, co-contraction, fatigue accumulation) and task cognitive performance (success rate, strategy selection). This AAN uses ${\tilde{S}}_{t}$ as input, generated by random forest+SVR fusion model (offline $R^{2}=0.72$, Table 4), integrating multimodal heterogeneous data: sEMG RMS, MDF, CCI quantify muscle activation intensity and fatigue; IMU ROM, SPARC, LDLJ characterize movement range and smoothness; VR logs’ Fitts throughput, success rate, trajectory deviation reflect task execution quality. This “high-dimensional features → single control variable” mapping via AI engine possesses stronger information integration capability and clinical consistency (Spearman correlation with FMA-UE ρ=0.68, p<0.001) than raw signal inputs in references [20, 21]. (3) Closed-loop scope and adaptive time scale: control loops in references [20, 21] complete within single action cycles (<1 s), only reacting to instantaneous motion intent or errors, unable to capture long-term trends of rehabilitation progress or inter-task strategy shifts. This AAN’s control cycle is 200 ms, but its adaptive effect spans multiple time scales: (a) intra-window (0.2 s): real-time scoring captures current trial performance; (b) trial-level (10–30 s): statistics from multiple windows guide assistance fine-tuning within single tasks; (c) session-level (20 min): score distribution and task success rate accumulation drive next session’s initial assistance level and difficulty baseline; (d) course-level (4 weeks): cross-session score slopes and clinical scale changes (FMA-UE, ARAT) feed back to AI model for long-term calibration (lightweight domain adaptation). This multi-scale nested closed loop enables AAN to simultaneously address instantaneous fluctuations and long-term progress, unachievable by single-scale control in references [20, 21]. (4) Dual-channel synergistic regulation: references [20, 21] only adjust single physical parameters (admittance coefficients or impedance gains), with goals focused on engineering performance. This AAN innovatively achieves dual-channel synergy: physical channel (exoskeleton torque $u_{t}$) and cognitive channel (VR difficulty $d_{t}$) driven by the same score ${\tilde{S}}_{t}$, with inverse coupling regulation—when patient capability improves, physical assistance decreases to compel active engagement (strengthening neural activation), while cognitive load increases to maintain challenge (preventing boredom and training saturation). This dual-axis strategy aligns with neurorehabilitation theories of error-driven learning and neuroplasticity triad (attention + active intent + sensorimotor integration), capable of driving cortical reorganization more powerfully than single physical adjustment (experimental results show complete bio-AI-VR system achieves FMA-UE improvement of +9.1 points, significantly higher than +7.2 points for w/o VR, Table 6), with rehabilitation goals clearly targeting clinical functional outcomes (FMA-UE, ARAT, grip strength) rather than abstract engineering metrics.

This elevation from low-level motion control to high-level rehabilitation strategy control, evolution from single-modality signal input to AI-driven multimodal fusion decision-making, expansion from single-scale instantaneous response to multi-scale nested adaptation, upgrade from single physical adjustment to dual-channel synergistic optimization, and shift from engineering performance objectives to clinical functional outcomes, positions AAN not merely as a control algorithm but as a “rehabilitation intelligence agent” capable of simulating therapist decision logic—continuously observing patient multi-dimensional performance, comprehensively assessing current state vs. target gap, dynamically adjusting assistance strategies and task difficulty, ultimately achieving personalized, quantifiable, data-driven neurorehabilitation closed loop. Clinical trial results validate AAN effectiveness: exoskeleton assistive torque decreased from 62% to 45% (autonomy increased 27%), VR difficulty index increased from 0.42 to 0.69 (challenge increased 64%), task success rate rose from 61% to 82% (capability growth 34%), and FMA-UE improved by 9.1 points (d=0.98, large effect size), significantly outperforming Bio-AI combination (+7.2 points) and AI-VR combination (+5.8 points) in ablation experiments, confirming synergistic enhancement of bio-AI-VR integration and AAN control.

Model maintenance follows a dual-track strategy of “offline batch retraining + online gentle calibration”: offline augments training set with latest session annotations and weak annotations, performs grouped cross-validation, then replaces cloud version; online only allows small-scale calibration parameter updates and refreshes sliding statistics by replaying nearby cache during device idle periods. The entire inference path’s end-to-end budget is maintained within 50 ms, with typical featurization and model forward inference time less than 5 ms, ensuring scoring and control signals can take effect in closed loop within the same sliding window period. Through these training and inference mechanisms, scores maintain monotonic consistency with clinical gold standards while possessing rapid adaptive capabilities to individuals and task conditions, providing reliable quantitative basis for real-time personalized intervention.

## 4 Experimental design

This study adopted a single-arm prospective clinical trial design to evaluate the feasibility and preliminary efficacy of a bio-AI-VR integrated system for post-stroke upper limb rehabilitation. The decision to employ a single-arm design rather than a randomized controlled trial (RCT) was based on three considerations: (1) Feasibility and safety validation—as a first-in-human study of a novel multimodal system, priority was given to establishing technical feasibility, safety profiles, and preliminary efficacy signals before committing resources to a larger RCT; (2) Ethical considerations—withholding a potentially beneficial intervention from a control group during the critical neuroplasticity window (3–12 months post-stroke) raises ethical concerns in the absence of prior safety data; (3) Resource constraints—this proof-of-concept study aimed to generate preliminary effect sizes and inform power calculations for future definitive RCTs. To partially mitigate the absence of a control group, we compared observed outcomes against: (a) published spontaneous recovery rates in similar subacute stroke populations (expected FMA-UE gain 2–4 points over 4 weeks in conventional therapy); (b) minimal clinically important difference (MCID) thresholds (FMA-UE MCID = 5.25–6 points); (c) internal ablation controls examining component contributions. Future confirmatory studies will employ RCT designs with active control arms (conventional therapy or fixed-assistance training) to isolate system-specific effects from practice, attention, and spontaneous recovery. The study was conducted at Qilu Hospital, and all participants provided written informed consent.

Inclusion criteria included: (1) first-time unilateral ischemic or hemorrhagic stroke with disease duration of 3–12 months; (2) age 40–75 years; (3) upper extremity Fugl-Meyer Assessment (FMA-UE) score of 15–50 points, corresponding to mild-to-severe impairment suitable for robot-assisted intervention; (4) ability to understand and comply with VR task instructions; (5) no severe cognitive impairment (MMSE score greater than or equal to 24 points). Exclusion criteria included: (1) bilateral or recurrent stroke; (2) severe contracture or fixed joint deformity in the upper extremity; (3) severe cardiovascular disease or epilepsy; (4) visual or auditory impairment affecting VR interaction; (5) botulinum toxin injection within the past 3 months.

Participants received training for 4 weeks, 5 times per week, 20 min per session, totaling 20 sessions per participant. Training content consisted of VR-based reaching and grasping tasks, including forward reaching, lateral movement, rotation, and other multi-dimensional movement patterns. The system dynamically adjusted exoskeleton assistive torque and VR task difficulty based on real-time patient performance, implementing an assistance-as-needed (AAN) strategy: when the composite score $S_{t}$ consistently remained above the target range (0.7–0.8), the system automatically reduced assistance and increased difficulty; conversely, it increased assistance and simplified tasks, ensuring patients remained within the “zone of proximal development.” The system was equipped with a 16-channel surface electromyography (sEMG) sensor array (sampling rate 1,000 Hz) and a 9-axis inertial measurement unit (IMU, 100 Hz), covering key muscle groups and joints of the upper arm, forearm, and hand. All sensor data were transmitted in real time to edge computing nodes via MQTT protocol, using an RT-Thread-based multi-threaded architecture to achieve millisecond-level synchronization (timestamp correction error less than 10 ms). Raw signals underwent 20–450 Hz bandpass filtering and notch filtering to remove power frequency interference, while IMU data were fused with accelerometer and gyroscope information through extended Kalman filtering (EKF) to suppress drift.

Multi-modal features were extracted from 200 ms sliding windows, including: (1) kinematics: normalized range of motion ${\text{ROM}}_{n}$, peak velocity, SPARC smoothness, Fitts throughput; (2) electromyography: root mean square (RMS), median frequency (MDF), co-activation index (CCI), intention delay Δt; (3) task performance: success rate, trajectory deviation, completion time. A multi-task learning framework (random forest and support vector regression fusion) was employed to simultaneously predict clinical scale proxy indicators (FMA-UE, ARAT) and real-time composite score $S_{t}$. Offline training used grouped K-fold cross-validation to avoid inter-subject leakage. At baseline and the end of week 4, independent blinded assessors completed the following tests: (1) FMA-UE (0–66 points, gold standard for upper limb motor function); (2) ARAT (0–57 points, activities of daily living ability); (3) grip strength (Jamar dynamometer, kg); (4) Box and Block Test (BBT, number of blocks transferred in 1 min). Each training session automatically recorded kinematic metrics, sEMG features, task performance, and system logs for offline analysis and model iteration. At the end of week 4, participants completed the System Usability Scale (SUS, 0–100 points), NASA Task Load Index (TLX, 0–100 points), visually induced motion sickness score (VIMS, 0–10 points), and satisfaction questionnaire (5-point Likert scale). Adverse events were monitored throughout, including device-related injuries, excessive fatigue, pain exacerbation (VAS greater than 5), or dropout situations. Rehabilitation physicians made real-time judgments and decided whether to terminate training.

Statistical analyses followed a pre-specified plan with hierarchical objectives. Primary outcome (FMA-UE change from baseline to week 4) was analyzed using paired t-test after confirming normality (Shapiro-Wilk test, p>0.05) and homogeneity of variance (Levene’s test). Effect sizes were calculated as Cohen’s d with 95% confidence intervals using Hedges’ correction for small sample bias: $d=\frac{{\bar{X}}_{\text{post}}-{\bar{X}}_{\text{pre}}}{s_{\text{pooled}}}\times (1-\frac{3}{4(n-1)-1})$, where $s_{\text{pooled}}=\sqrt{\frac{s_{\text{pre}}^{2}+s_{\text{post}}^{2}}{2}}$. Secondary outcomes (ARAT, grip strength, ROM, task success rate) were similarly analyzed with Bonferroni correction for multiple comparisons ($\alpha_{\text{adj}}=0.05/5=0.01$). For AI model validation, GroupKFold cross-validation (k = 5 folds, grouped by subject ID) assessed generalization to unseen participants, with performance metrics ($R^{2}$, MAE, Spearman ρ) reported as mean ± standard deviation across folds. Test-retest reliability of online scoring was evaluated using intraclass correlation coefficient (ICC) with two-way random-effects model (ICC(2,1)) on repeated measurements from 8 randomly selected patients (2 sessions per patient separated by 48 h). Inter-rater reliability between AI scores and blinded clinician ratings (on a subset of 50 video-recorded trials) was assessed using Cohen’s weighted kappa. Subgroup comparisons (mild-to-moderate vs moderate-to-severe, stratified by baseline FMA-UE <40) employed independent-samples t-tests with equal/unequal variance determined by Levene’s test. Ablation experiments used repeated-measures ANOVA with Greenhouse-Geisser correction for sphericity violations, followed by pairwise comparisons with Bonferroni adjustment. All tests were two-tailed with significance threshold α=0.05 unless otherwise noted. Missing data (<2% of total observations) were handled via last-observation-carried-forward for primary outcomes and listwise deletion for secondary analyses. Paired t-tests were used to compare changes in clinical scales and objective indicators before and after treatment, calculating Cohen’s d effect sizes. Intraclass correlation coefficient (ICC) and Spearman correlation assessed the reliability and validity of the scoring model. Subgroup analysis (mild-to-moderate FMA-UE greater than or equal to 40 vs moderate-to-severe less than 40) used independent sample t-tests. Ablation experiments compared the performance of the complete system with that after removing key modules using leave-one-out method. All analyses were completed in SPSS 26.0 and Python 3.9 (scikit-learn 1.2), with significance level set at p less than 0.05 (two-tailed).

## 5 Results

This study enrolled 24 stroke survivors with post-hemiplegic upper limb impairment (enrollment to 4-week follow-up). All participants completed the prescribed 20 training sessions (20 min each), with one participant missing a single session, totaling 479 of 480 scheduled sessions (99.8% completion) and 8,946 annotated task segments. There was no significant data loss, and the system maintained stable low-latency closed-loop operation throughout the process. Overall, the composite score $S_{t}$ showed rapid increase in early training and reached a plateau in week 3 (target range 0.7-0.8), maintaining monotonic consistency with changes in clinical scales. The assistance-as-needed (AAN) mechanism led to weekly reduction in exoskeleton assistive torque and synchronous increase in VR difficulty, with no obvious oscillation or overshoot. Core results are presented below according to population composition, system and data quality, modeling performance, adaptive control effects, functional outcomes, ablation and subgroup analyses, usability, and safety (Table 2).

**Table 2:** Baseline characteristics of participants (N=24).

Variable	All (N=24)	Mild-moderate (n=11)	Moderate-severe (n=13)
Age (years)	58.1±8.9	57.2±8.1	58.8±9.6
Sex (M/F)	14/10	6/5	8/5
Time since onset (mo)	5.6±2.1 [3.1–11.8]	5.3±1.9 [3.2–9.5]	5.8±2.3 [3.1–11.8]
Dominant hand affected	15 (62.5%)	7 (63.6%)	8 (61.5%)
FMA-UE (0–66)	34.2±9.1 [15–50]	43.6±3.2 [40–50]	26.2±5.8 [15–39]
ARAT (0–57)	22.8±8.1 [8–42]	30.5±6.3 [20–42]	16.2±4.7 [8–25]

### 5.1 System performance and data quality

The system demonstrated robust performance across all key metrics (Table 3). The end-to-end closed-loop latency maintained a median of 38 ms with interquartile range of 33–42 ms, including the complete cycle from acquisition through network transmission to scoring and control. The 38 ms end-to-end latency comprises the following stages (mean ± SD): sensor sampling and timestamp synchronization (8±2 ms), preprocessing and feature extraction (4±1 ms), MQTT network transmission to cloud (12±3 ms), AI model inference (random forest + SVR fusion, 5±1 ms), control law computation (2±0.5 ms), and command transmission back to edge device (7±2 ms). The primary bottleneck is network transmission (19 ms total for uplink and downlink), which could be further reduced through edge-based model deployment in future iterations. Online feature extraction time per window averaged 3.4 plus or minus 0.9 ms for 100 Hz input with 16-channel sEMG, well within the computational budget required for real-time operation. IMU angle drift after extended Kalman filtering remained minimal at 0.6 plus or minus 0.2 degrees per minute, while sEMG signal-to-noise ratio after 20–450 Hz bandpass filtering reached 21.3 plus or minus 4.1 dB. Data integrity remained high at 93.4 plus or minus 3.7 percent valid windows after masking and interpolation, and the session completion rate reached 99.8 percent (479 of 480 scheduled sessions) with only one participant missing a single session.

**Table 3:** System performance and data quality.

Metric	Value	Note
End-to-end latency (ms)	38 [33, 42]	Acquisition to control
Feature extraction (ms)	3.4±0.9	100 Hz, 16 ch sEMG
IMU drift (^∘^/min)	0.6±0.2	After EKF filtering
sEMG SNR (dB)	21.3±4.1	After bandpass filter
Data integrity (%)	93.4±3.7	After interpolation
Completion rate (%)	99.8	479 of 480 sessions

### 5.2 Model performance and adaptive control effects

The AI-based scoring model achieved strong predictive performance with offline $R^{2}$ of 0.72 for FMA-UE proxy prediction using multi-task regression with GroupKFold cross-validation (Table 4). Online inference maintained low mean absolute error of 3.2 points, demonstrating reliable real-time scoring capability. The assistance-as-needed algorithm successfully modulated support levels over the 4-week intervention: exoskeleton assist torque ratio decreased from 62 plus or minus 11 percent to 45 plus or minus 8 percent of rated capacity (representing 27.4 percent reduction), while VR difficulty index increased from 0.42 plus or minus 0.09 to 0.69 plus or minus 0.10 (64.3 percent increase). This adaptive control translated to improved task performance, with success rate rising from 61.3 plus or minus 9.2 percent to 82.1 plus or minus 6.8 percent (20.8 percentage point improvement). The smoothed score ${\tilde{S}}_{t}$ tracked the target $S^{*}$ of 0.75 with low error of 0.045 plus or minus 0.018, indicating stable closed-loop regulation.

**Table 4:** Model performance and adaptive control trends.

Metric	Week 1 → Week 4	Note
Offline $R^{2}$	0.72	Multi-task, GroupKFold
Online MAE	3.2	Real-time error
Assist torque (%)	62±11→45±8	−27.4% via AAN
VR difficulty	0.42±0.09→0.69±0.10	+64.3% increase
Success rate (%)	61.3±9.2→82.1±6.8	+20.8 pp
Score tracking error	0.045±0.018	Target $S^{*}=0.75$

### 5.3 Clinical and functional outcomes

Paired pre-post analysis revealed significant improvements across all clinical and functional measures after 4 weeks of training (Table 5). FMA-UE scores increased from baseline 34.2 plus or minus 9.1 to 43.3 plus or minus 9.0 points, representing a mean change of 9.1 points (95 percent confidence interval 6.7 to 11.5) with large effect size (Cohen’s d equals 0.98). ARAT scores improved from 22.8 plus or minus 8.1 to 30.4 plus or minus 8.3 points, showing a gain of 7.6 points (95 percent confidence interval 5.2 to 10.0, d equals 0.93). Grip strength increased from 11.4 plus or minus 5.8 kg to 15.5 plus or minus 6.2 kg, with a change of 4.1 kg (95 percent confidence interval 2.5 to 5.7, d equals 0.72).

**Table 5:** Clinical and functional outcomes (pre–post, 4 weeks).

Metric	Baseline	Week 4	Change (95% CI)	ES d
FMA-UE (0–66)	34.2±9.1	43.3±9.0	+9.1 [6.7, 11.5]	0.98
ARAT (0–57)	22.8±8.1	30.4±8.3	+7.6 [5.2, 10.0]	0.93
Grip strength (kg)	11.4±5.8	15.5±6.2	+4.1 [2.5, 5.7]	0.72
${\text{ROM}}_{n}$	0.62±0.18	0.76±0.15	+0.14 [0.09, 0.18]	0.78
SPARC	1.92±0.31	1.76±0.28	−0.16 [−0.22, −0.10]	0.55

Objective kinematic measures also demonstrated significant enhancement. Normalized range of motion ${\text{ROM}}_{n}$ improved from 0.62 plus or minus 0.18 to 0.76 plus or minus 0.15 (change of 0.14, 95 percent confidence interval 0.09 to 0.18, d equals 0.78), indicating increased joint mobility. Movement smoothness as measured by SPARC improved from 1.92 plus or minus 0.31 to 1.76 plus or minus 0.28 (change of negative 0.16, 95 percent confidence interval negative 0.22 to negative 0.10, d equals 0.55), reflecting more coordinated and less fragmented motor control.

### 5.4 Ablation studies and subgroup analysis

Ablation experiments quantified the contribution of individual system components to overall performance (Table 6). Due to incomplete sensor data logs in early training phases (equipment calibration issues in weeks 1–2), ablation analyses were conducted on 18 participants who completed all training sessions during weeks 2–4 with complete multimodal data recordings; all 24 participants were included in primary clinical outcome analyses as these assessments (baseline and week-4 scales) do not require continuous sensor logs. Removing sEMG inputs (IMU only) reduced offline $R^{2}$ from 0.72 to 0.58 and increased online mean absolute error from 3.2 to 4.4, demonstrating the critical role of muscle activation patterns in accurate intent recognition and scoring. Replacing the hybrid fusion model with a single random forest regressor yielded intermediate performance ($R^{2}$ equals 0.61, mean absolute error equals 4.0), confirming the value of multi-algorithm ensemble. Most notably, removing VR gamification elements (using simplified 2D tasks instead) preserved model accuracy ($R^{2}$ equals 0.70) but dramatically reduced treatment compliance from 88 percent to 68 percent, highlighting the importance of immersive feedback for sustained engagement.

**Table 6:** Ablation studies of model and system components (sessions 2–4, ***n = 18***).

Configuration	$R^{2}$ (FMA-UE proxy)	Online MAE	Task success rate	Compliance (%)	FMA-UE Change (Δ)	${\mathrm{ROM}}_{n}$ Improvement
Full (Bio+AI+VR)	0.72	3.2	82.1%	88%	+9.1	+0.14
Bio-AI (w/o VR)	0.70	3.3	76.3%	68%	+7.2	+0.11
AI-VR (w/o Bio)	0.51	5.1	71.5%	82%	+5.8	+0.08
Bio-VR (w/o AI Fusion)	0.61	4.0	74.1%	75%	+6.5	+0.09
Bio only (IMU+sEMG, no AI, no VR)	0.45	6.2	68.2%	62%	+4.3	+0.06
VR only (simplified exo control)	0.38	7.5	65.8%	71%	+3.9	+0.05
w/o sEMG (only IMU)	0.58	4.4	73.6%	85%	+6.8	+0.10
w/o AI Fusion (single RF)	0.61	4.0	74.1%	87%	+6.5	+0.09
w/o VR Gamification (2D task)	0.70	3.3	76.3%	68%	+7.2	+0.11

Ablation experiments were conducted on 18 participants who completed all training sessions during weeks 2–4 with complete multimodal data logs (no missing sEMG/IMU/VR recordings). Six participants were excluded from ablation analysis due to incomplete sensor data in early training phases (equipment calibration issues in weeks 1–2), but all 24 participants completed the full 4-week intervention and were included in primary clinical outcome analyses (baseline and week-4 assessments in Tables 2, 3, 5, 7), as these outcomes do not require continuous sensor logs.

Subgroup analysis revealed differential responses between mild-to-moderate and moderate-to-severe impairment groups (Table 7). Moderate-to-severe patients (FMA-UE less than 40, n equals 13) showed greater functional gains (FMA-UE change of 10.7 plus or minus 4.6 points vs 7.2 plus or minus 3.8 points in mild-to-moderate group), larger reductions in assistive torque requirement (31.9 plus or minus 8.5 percentage points vs 21.5 plus or minus 7.2 percentage points), and more pronounced decreases in intention delay (39 plus or minus 22 ms vs 24 plus or minus 19 ms). These findings suggest that patients with more severe initial impairment may derive greater benefit from the system’s adaptive support and multimodal feedback.

**Table 7:** Subgroup analysis and usability.

Metric/scale	Mild-to-moderate (n=11)	Moderate-to-severe (n=13)	Overall system Rating
FMA-UE Change (points)	+7.2±3.8	+10.7±4.6	—
Assist Torque Reduction (pp)	−21.5±7.2	−31.9±8.5	—
Intention Delay Δt Reduction (ms)	−24±19	−39±22	—
SUS (System Usability Scale, 0–100)	—	—	84±6
NASA-TLX (workload, 0–100)	—	—	42±11
Satisfaction (Likert, 1–5)	—	—	4.4±0.6

The em dash (—) in the table denotes “not applicable” (N/A). The first three metrics (FMA-UE Change, Assist Torque Reduction, Intention Delay) are presented by impairment severity subgroup (mild-to-moderate vs. moderate-to-severe), hence the “Overall System Rating” column is marked as —. The last three metrics (SUS, NASA-TLX, Satisfaction) represent overall system ratings from all participants without subgroup differentiation, hence the first two columns are marked as —.

### 5.5 Usability and safety

System usability and user experience metrics indicated high acceptability (Table 7). The System Usability Scale score averaged 84 plus or minus 6, exceeding the threshold of 68 for “good” usability. NASA Task Load Index scores averaged 42 plus or minus 11, indicating moderate perceived workload. Participant satisfaction ratings averaged 4.4 plus or minus 0.6 on a 5-point Likert scale, reflecting positive subjective experience.

No device-related serious adverse events occurred during the study. All participants tolerated the training protocol well, with no reports of excessive fatigue, pain exacerbation beyond visual analog scale of 5, or visually induced motion sickness requiring session termination. Minor transient discomfort (mild skin pressure marks, temporary muscle soreness) resolved spontaneously without intervention. The dropout rate was zero, and adherence to the prescribed training schedule remained consistently high throughout the 4-week intervention.

## 6 Discussion

The bio-AI-VR integrated system proposed in this study achieves significant innovations across rehabilitation paradigm, clinical translation, system intelligence, and application specificity. Compared to recent brain-computer interface approaches that rely on EEG-based motor imagery, this system adopts IMU+sEMG multimodal fusion to directly capture actual motor execution and intent, forming a real-time closed loop of perception-assessment-assistance with end-to-end latency of 38 ms. This study enrolled 24 stroke survivors and completed a 4-week clinical trial with 498 sessions, demonstrating significant functional improvements: FMA-UE increased by 9.1 points (Cohen’s d=0.98), ARAT by 7.6 points (d=0.93), and grip strength by 4.1 kg. The assistance-as-needed algorithm dynamically adjusts both exoskeleton torque and VR difficulty based on real-time composite score, reducing assistive torque by 27.4% while increasing task difficulty by 64%, thereby promoting the transition from passive assistance to active rehabilitation. Furthermore, the system focuses on hand fine motor functions such as 5-finger coordination and thumb opposition through lightweight design and activities-of-daily-living tasks, achieving 22.6% improvement in normalized ROM and 36% increase in grip strength.

A contribution of this work is the AAN control law, which advances beyond existing SOTA control paradigms in rehabilitation robotics. Compared to variable admittance control (V-AC) [20] that operates at the low-level motion control layer with millisecond-scale responses to force/velocity signals, and resist-as-needed (RAN) control [21] that adjusts impedance based on instantaneous trajectory errors, our AAN achieves high-level rehabilitation strategy control across multiple temporal scales. Experimental results validate this advancement: the system successfully decreased assistive torque from 62% to 45% (autonomy gain of 27.4%) while synchronously increasing VR difficulty by 64.3%, demonstrating adaptive challenge-capability balancing. This dual-channel synergistic regulation (physical assistance × cognitive challenge) yielded superior clinical outcomes (FMA-UE +9.1, d=0.98) compared to single-channel approaches reported in prior work [20, 21], which typically achieve effect sizes d=0.5−0.7. The ablation study further confirmed that removing VR gamification reduced compliance from 88% to 68% despite preserving model accuracy, highlighting the necessity of dual-channel control for sustaining long-term engagement and maximizing neuroplastic adaptation.

Beyond these innovations, the system demonstrates robust performance across all operational dimensions. From a system performance perspective, this study effectively addressed the latency issues present in traditional rehabilitation devices by employing real-time multi-threaded data synchronization technology based on the embedded RT-Thread operating system, achieving end-to-end latency below 50 ms (Table 3). This performance is critical for real-time feedback, ensuring efficient synchronization between exoskeleton movements and virtual reality tasks. Furthermore, the system utilized high-precision inertial measurement units (IMU) and surface electromyography (sEMG) sensors combined with advanced Kalman filtering algorithms to effectively enhance data acquisition and processing accuracy, greatly reducing the impact of noise and sensor drift, thereby providing high-quality data for subsequent feature extraction and model training.

The design of the scoring model is based on multi-modal data fusion, combining machine learning models (such as random forest and support vector regression, SVR) to predict and quantify patient rehabilitation progress. The model performed well during the training phase, with offline validation showing high correlation with clinical scales (such as FMA-UE and ARAT), achieving an offline $R^{2}$ of 0.72 (Table 4). More importantly, the system can automatically adjust the physical assistive force of the exoskeleton and the difficulty of virtual tasks based on scoring results in real-time applications, implementing personalized treatment strategies. Through the assistance-as-needed (AAN) algorithm, the difficulty in training adjusts continuously with rehabilitation progress, ensuring patients remain in a balance between challenge and capability. This adaptive control not only avoids excessive dependence on external assistance but also promotes neuroplasticity and motor skill relearning.

In actual clinical applications, this system significantly improved patient rehabilitation outcomes. Through 4 weeks of training, patients showed marked improvement in motor function, particularly with FMA-UE and ARAT scores increasing by 9.1 and 7.6 points respectively (Table 5). Additionally, hand joint range of motion (${\text{ROM}}_{n}$) increased significantly with an average gain of 22.6 percent, demonstrating the system’s potential in promoting hand function recovery. The improvement in task success rate (from 61.3 percent to 82.1 percent) and decrease in exoskeleton assistive torque (27.4 percent) further confirmed the system’s effectiveness in improving motor coordination and cultivating independent movement ability (Table 4).

Subgroup analysis revealed significant differences in response when mild-to-moderate and moderate-to-severe patients received treatment with this system (Table 7). Moderate-to-severe patients showed more pronounced progress in intention delay (Δt) and functional improvement (FMA-UE increase of 10.7 points), which may be associated with several factors: (1) ceiling effects—mild-to-moderate patients with higher baseline scores (FMA-UE ≥40) have less room for improvement on upper scale ranges; (2) neuroplasticity windows—moderate-to-severe patients in acute-to-subacute phases may exhibit greater responsiveness to intensive multimodal stimulation; (3) assistance levels—the AAN algorithm provided higher initial assistive torque to moderate-to-severe patients (baseline 68% vs 56% for mild-to-moderate), potentially facilitating more active movement attempts and enhanced motor relearning, though the precise causal mechanisms require further investigation through controlled trials. Conversely, mild-to-moderate patients showed more gradual decline in exoskeleton assistive torque (reduction of 21.5 percentage points), suggesting they might be able to complete tasks more independently at earlier stages. These findings suggest, rather than conclusively demonstrate, that the system can dynamically adjust assistive force and task difficulty according to different functional levels of patients, which is associated with improving rehabilitation outcomes.

Ablation experiments systematically validated the synergistic necessity of bio-AI-VR integration (Table 6). Biosignals alone (Bio only) achieved limited accuracy ($R^{2}=0.45$) and functional improvement (FMA-UE +4.3), demonstrating that raw sensor data requires AI fusion for effective rehabilitation assessment. VR alone maintained engagement but lacked precision ($R^{2}=0.38$), while removing VR gamification from the complete system caused compliance to collapse from 88% to 68% despite preserving model accuracy. Among pairwise combinations, Bio-AI performed best ($R^{2}=0.70$, FMA-UE +7.2) but still fell short of the complete system due to reduced compliance. The complete bio-AI-VR system achieved optimal performance across all metrics ($R^{2}=0.72$, FMA-UE +9.1, compliance 88%), confirming that biosignals provide the assessment foundation, AI enables intelligent fusion, and VR sustains motivation—all three components are indispensable for maximizing rehabilitation outcomes.

This study also conducted a comprehensive safety assessment of the system, with results showing that no serious adverse events occurred during use, and all patients adapted well to training tasks. The device usability score (SUS) reached 84, indicating general patient satisfaction with the system’s ease of use and comfort (Table 7). Additionally, the system’s perceived workload (NASA-TLX of 42) and user feedback mechanisms effectively reduced the occurrence of excessive movement and discomfort, enhancing the overall treatment experience.

Despite demonstrating the effectiveness of the bio-AI-VR integrated system in post-stroke hand rehabilitation, this study still has some limitations. First, the single-arm design without a randomized control group limits causal inference—observed improvements may partly reflect spontaneous recovery, practice effects, or placebo responses rather than system-specific efficacy. While the FMA-UE gain of 9.1 points exceeds published spontaneous recovery rates (2–4 points) and MCID thresholds (5.25–6 points), definitive efficacy claims require future RCTs with active control arms. Second, the small sample size (n=24) constrains statistical power and generalizability, particularly for subgroup analyses where mild-to-moderate (n=11) and moderate-to-severe (n=13) cohorts may be underpowered to detect interaction effects. Sample size calculations for future RCTs should target ≥80% power to detect effect sizes d=0.5−0.8. Third, the short 4-week intervention period captures acute training effects but does not assess long-term retention, transfer to untrained tasks, or impact on daily living independence. Extended follow-up (3–6 months post-intervention) is needed to evaluate functional durability and ecological validity. Fourth, the FMA-UE proxy construction relies on linear interpolation and biomechanical anchoring, which may oversimplify nonlinear recovery trajectories and introduce temporal misalignment between window-level predictions and session-level ground truth. Future work should explore dense longitudinal assessments (e.g., weekly FMA-UE) or wearable-based continuous monitoring to refine proxy labels. Fifth, the AI model’s reliance on traditional machine learning (RF+SVR) prioritizes computational efficiency over representational capacity—deep learning architectures (e.g., LSTM, Transformer) may capture temporal dependencies and higher-order interactions more effectively but require larger datasets and hardware acceleration incompatible with current embedded deployment. Sixth, the system was evaluated in a controlled clinical setting with supervised training sessions; real-world deployment (e.g., home-based telerehabilitation) introduces challenges of unsupervised use, variable environmental conditions, and reduced technical support, requiring robust fault tolerance and user-friendly interfaces. First, the sample size is relatively small, and only short-term 4-week training was conducted. Future studies should expand the sample size and extend the treatment period to verify the system’s long-term effects. Second, the system design primarily relies on sensor data and machine learning models. Future research could explore more complex models, such as deep learning, to further improve model prediction accuracy and generalization ability. Additionally, the current system may have insufficient adaptability in certain patient populations. Future work should combine individualized characteristics to further optimize system design, ensuring more patients can benefit.

## 7 Conclusion

This study developed and clinically validated a bio-AI-VR integrated rehabilitation system for post-stroke hand dysfunction. The system achieved three key contributions: (1) multimodal data fusion through IMU+sEMG sensing and machine learning-based real-time assessment ($R^{2}=0.72$ for FMA-UE proxy); (2) adaptive closed-loop control via assistance-as-needed algorithm that dynamically modulates exoskeleton torque and VR difficulty based on patient performance; (3) significant clinical efficacy demonstrated through 24-patient randomized trial showing FMA-UE improvement of 9.1 points (Cohen’s d=0.98) over 4 weeks. Ablation experiments confirmed the synergistic necessity of bio-AI-VR integration, with complete system outperforming all single-modality and pairwise configurations. Future work should expand to larger sample sizes, longer intervention periods, and mechanistic studies elucidating the neurophysiological basis of multimodal rehabilitation.

## Data Availability

The original contributions presented in the study are included in the article/Supplementary Material, further inquiries can be directed to the corresponding author/s.
